# Clinical development of nitisinone for alkaptonuria (developakure) - a rare disease clinical trials design

**DOI:** 10.1186/1745-6215-14-S1-P29

**Published:** 2013-11-29

**Authors:** Eftychia Eirini Psarelli, Trevor Cox, Lakshminarayan Ranganath

**Affiliations:** 1 University of Liverpool, Liverpool, Merseyside, UK; 2 Royal Liverpool University Hospital, Liverpool, Merseyside, UK

## 

Alkaptonuria (AKU) is an orphan inherited homogentisate dioxygenase enzyme deficiency resulting in accumulation of homogentisic acid (HGA). HGA is converted to a black pigment polymer known as ochronosis that causes tissue damage affecting many tissues including joints and heart, with significant poor quality of life. The DevelopAKUre project is a Europe-wide collaboration to study the efficacy and safety of nitisinone as a potential treatment in three clinical studies. The first is a phase 2 dose-response study (SONIA 1) which will determine the optimal dose of nitisinone that decreases HGA levels. The second is a phase III efficacy study (SONIA 2) based on the optimal dose and the third is a cross-sectional study (SOFIA) that will define the age that treatment should begin after determining the onset of ochronosis. Details and rationale of the SONIA 2 design will be described, with special attention to issues arising from the rarity of the disease.

